# Evaluation of pre-analytical specimen rejection using Six Sigma metrics: A retrospective single-center study

**DOI:** 10.1371/journal.pone.0324840

**Published:** 2025-06-03

**Authors:** Xiuli Cheng, Haimiao Yu, Le Zhang, Biao Zhang, Qin Wang

**Affiliations:** 1 Department of Clinical Laboratory, Tianjin Key Laboratory of Cerebral Vessels and Neural Degeneration, Tianjin Huanhu Hospital, Tianjin, China; 2 Huanhu Hospital Affiliated to Tianjin Medical University, Tianjin, China; Versiti Blood Research Institute, UNITED STATES OF AMERICA

## Abstract

**Background:**

Up to 60% of errors occur in the pre-analytical stage of laboratory testing, potentially impairing clinical decision-making. This study aimed to assess pre-analytical errors using Six Sigma metrics and identify underlying causes for quality improvement.

**Methods:**

A retrospective analysis of pre-analytical sample errors was conducted over three years in a clinical laboratory. Errors were categorized, and Sigma values were calculated to assess quality. Trends over time were also analyzed.

**Results:**

Of 2,068,074 samples, 2,214 (0.107%) were rejected. The top errors were clotted blood specimens (67.34%), insufficient volumes (8.22%), and cancelled test requests (6.28%), with Sigma values of 4.42, 5.25, and 5.32, respectively. The outpatient department performed best (Sigma = 5.47), while other wards required improvement.

**Conclusion:**

Efforts are needed to reduce specimen rejection, particularly clotted samples, to enhance laboratory quality.

## Introduction

Quality is the backbone and main issue of all laboratories. However, more than 60% of errors occurred in the pre-analytical stage of laboratory testing and may impair the clinical decision-making process [1,2]. The pre-analytical phase comprises all events from the time a test requisition made by a physician until the time the sample analyzed at the lab. Generally, it includes test requisition, patient preparation, sample collection, storage and transportation, which is usually outside the direct control of laboratory, thus requires special attention.

Six Sigma metrics were originally developed by the telecommunications company Motorola in the early 1980s as a robust framework for enhancing quality management. A Six Sigma level, equivalent to 3.4 defects per million opportunities (DPMO), is recognized as world-class quality, while a 3-Sigma level, with 66,807 DPMO, is considered the minimum acceptable standard. These metrics provide a quantitative and standardized approach to evaluating process performance, enabling organizations to identify and reduce variability, improve efficiency, and achieve sustained quality improvement.

In the context of laboratory testing, Six Sigma metrics can be applied to assess the quality of pre-analytical, analytical, and post-analytical processes [3]. For the pre-analytical phase, errors are quantified as DPMO, which can then be converted into Sigma values to objectively evaluate laboratory performance [4,5]. This approach allows laboratories to pinpoint specific areas of weakness, implement targeted improvements, and monitor progress over time. By adopting Six Sigma principles, laboratories can move beyond traditional error rate percentages and adopt a more rigorous, data-driven methodology to enhance overall quality and patient outcomes.

In this study, we performed a retrospective analysis of specimen rejection data spanning a three-year period in a clinical laboratory. The causes and sources of sample rejection were systematically investigated, and errors were quantified using Six Sigma metrics to assess the quality of the pre-analytical phase. Furthermore, temporal trends in error rates were analyzed to identify potential systemic issues and guide the development of targeted corrective strategies for sustainable quality improvement. As one of the first studies to apply Six Sigma metrics to evaluate pre-analytical errors in a specialized brain hospital, this research offers valuable insights into optimizing laboratory workflows and advancing overall healthcare quality.

## Materials and methods

### Criteria for sample rejection

This study was conducted in the clinical laboratory of Tianjin Huanhu Hospital, a tertiary care hospital specializing in brain-related conditions. We retrospectively analyzed all samples received between January 2021 and December 2023, excluding COVID-19 specimens due to their distinct handling protocols. The laboratory had established predefined rejection criteria for samples with pre-analytical errors.

Clotted, hemolyzed, icteric, and lipemic samples were visually assessed by two technologists. Clotted samples were specifically evaluated in anticoagulated blood specimens. A minimum volume requirement was established based on methodological and automation standards; however, samples with volume errors that resulted in improper blood-to-anticoagulant ratios for coagulation tests were uniformly rejected. Additionally, test requests with incomplete, illegible, or inaccurate information were also rejected.

### Data collection and statistical analysis

All samples were transported to the laboratory via an air tube transport system. Upon arrival, technicians conducted an initial inspection to identify any errors. Samples meeting the predefined rejection criteria were promptly rejected, and a request for new sample collection was initiated. Simultaneously, detailed information regarding the errors was recorded in the Laboratory Information System (LIS).

For data analysis, error records were extracted from the LIS and categorized according to the rejection criteria. The frequencies of each error type were calculated using Microsoft Office Excel, and the Sigma values were calculated by converting the observed error rates (or defect rates) using a standard Sigma conversion table, as described by Harry and Schroeder [6]. This method is based on the relationship between defect rates, DPMO and Sigma levels, which is widely used in Six Sigma quality management. The standard conversion tables used for calculating Sigma values are provided in the Supporting Information S1 Table.

To assess the variation in error rates among different clinical wards, we performed hierarchical clustering (Ward’s method) on hospital wards using standardized error rates and Sigma values. Cluster validity was confirmed by significant between-group differences (Kruskal-Wallis, *p* < 0.05) and within-group homogeneity (Levene’s, *p* > 0.10). Statistical comparisons were performed using one-way ANOVA with Fisher’s Least Significant Difference (LSD) post hoc tests for multiple comparisons. A paired Wilcoxon signed-rank test was implemented to statistically compare the prevalence rate of clotted samples against the aggregate prevalence of all other pre-analytical error categories. All statistical data were analyzed by the SPSS 26.0 software and GraphPad Prism 5. *P* < 0.05 was considered statistically significant.

### Ethics statement

This study was approved by the Ethics Committee of Tianjin Huanhu Hospital (Ethical Approval No.2024−149). The ethics committee waived the requirement for informed consent because the research involved fully anonymized data derived from rejected specimens’ records and all direct identifiers (e.g., name, patient ID) and indirect identifiers (e.g., rare diagnoses) were removed prior to analysis, ensuring no re-identification risk. Authors had no access to information that could identify individual participants during or after data collection. The study posed minimal risk to participants as it focused solely on pre-analytical quality metrics without affecting clinical care. The data were accessed for research purposes at July 2, 2024.

## Results

### General characteristics of specimen rejection

All requests with errors were rejected during the study period. Of the 2,068,074 samples received between January 2021 and December 2023, 2,214 samples (0.107%) were rejected due to various factors that could potentially compromise test results. The rejection reasons were further categorized, as detailed in Table 1.

**Table 1:** Distribution of pre-analytical errors and Sigma values (N = 2,068,074).

S. No.	Pre-analytical errors	Number of errors (percentage)	Error rate (errors/N × 100)	Defects per million opportunities	Sigma value
1	Improper test requests (incomplete, illegible, inaccurate information)	61 (2.76%)	0.002950	29.50	5.52
2	Inappropriate transport (delayed transport time, transport temperature)	5 (0.23%)	0.000242	2.42	6.0
3	Incorrect container or tube	32(1.45%)	0.001547	15.47	5.67
4	Insufficient specimen volume or improper ratio of blood to anticoagulant	182 (8.22%)	0.008800	88.00	5.25
5	Hemolyzed/lipemic samples	117 (5.28%)	0.005657	56.57	5.36
6	Request without specimen/duplicate specimens	50 (2.26%)	0.002418	24.18	5.56
7	Partially/fully clotted samples	1491(67.34%)	0.173547^a^	1735.47^a^	4.42
8	Contaminated by intravenous infusion	66 (2.98%)	0.003191	31.91	5.50
9	Spilled specimens	23 (1.04%)	0.001112	11.12	5.74
10	Test requests cancelled	139(6.28%)	0.006721	67.21	5.32
11	Draw the wrong patient	26(1.17%)	0.001257	12.57	5.71
12	Lost samples	0 (0%)	0	0	∞
13	Other reasons	22 (0.99%)	0.001064	10.64	5.75
	Total	2214 (100%)	0.107056	1070.56	4.57

^a^This value referred to the ratio of clotted samples (1491) to the total anticoagulant blood samples (859,131).

Among the rejected samples, 1,491 (67.34%) were partially or fully clotted, representing the most common cause of rejection. Insufficient specimen volume, including improper blood-to-anticoagulant ratios, accounted for the second highest proportion of rejections (8.22%), followed by cancelled test requests (6.28%). The Sigma values for these top three causes were 4.42, 5.25, and 5.32, respectively. Additionally, hemolyzed or lipemic samples constituted a significant proportion of rejections (5.28%), ranking just below cancelled test requests. Notably, no specimens were lost during the investigated period.

### Distribution of the rejected specimens

**Fig 1:**
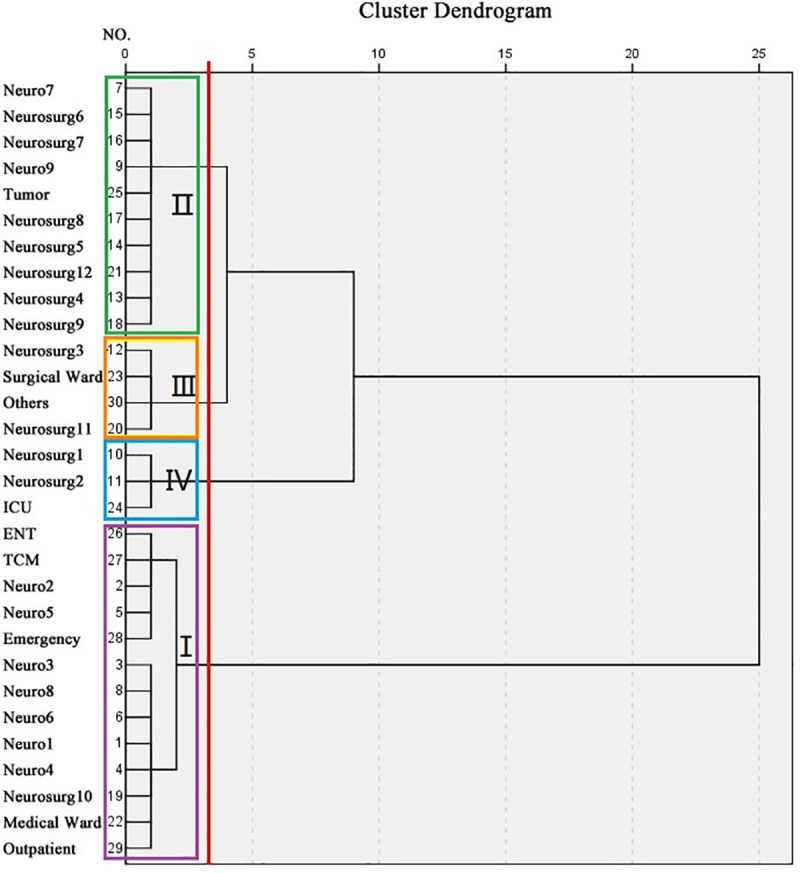
Hierarchical Clustering Analysis of Error Rates across Hospital Wards. Hierarchical clustering dendrogram of clinical departments based on specimen rejection patterns, using Ward’s linkage method with Euclidean distance. The analysis identified four distinct clusters (labeled I-IV). Cutoff at linkage distance = 3.5 (red line) determined the optimal cluster solution. Abbreviation: Neuro, neurology; Neurosurg, neurosurgery; ENT, department of eye, otolaryngology, head and neck surgery; TCM, traditional Chinese medicine.

To identify areas requiring quality improvement interventions, we performed a hierarchical cluster analysis of specimen rejection rates and Sigma values across hospital departments. As shown in Table 2, the rejection rates and corresponding Sigma values varied among different wards. The analysis revealed four distinct performance clusters (Table 3 and Fig 1), with statistically significant inter-group differences (Kruskal-Wallis χ² = 25.5, *p *< 0.001).

**Table 2:** Distribution and Sigma values of specimen rejection from each department.

Departments		Number of errors	Total samples	Error rate (errors/N × 100)	Defects per million opportunities	Sigma value
Department of Neurology	Ward 1	70	76298	0.091745	917.4544	4.62
	Ward 2	66	62344	0.105865	1058.647	4.57
	Ward 3	31	52325	0.059246	592.4558	4.74
	Ward 4	79	82043	0.096291	962.9106	4.60
	Ward 5	78	72095	0.108191	1081.911	4.57
	Ward 6	29	55151	0.052583	525.826	4.78
	Ward 7	102	76134	0.133974	1339.741	4.50
	Ward 8	27	46534	0.058023	580.2258	4.75
	Ward 9	84	50803	0.165344	1653.445	4.44
Neurosurgery ward	Ward 1	158	39129	0.403797	4037.97	4.15
	Ward 2	153	35350	0.432809	4328.089	4.13
	Ward 3	56	25675	0.218114	2181.139	4.35
	Ward 4	59	37738	0.15634	1563.4	4.46
	Ward 5	65	42052	0.154571	1545.712	4.46
	Ward 6	53	39469	0.134282	1342.815	4.5
	Ward 7	38	27240	0.139501	1395.005	4.49
	Ward 8	45	27839	0.161646	1616.455	4.44
	Ward 9	38	25442	0.149362	1493.622	4.47
	Ward 10	25	31829	0.078544	785.4365	4.66
	Ward 11	97	36388	0.266571	2665.708	4.29
	Ward 12	15	9810	0.152907	1529.068	4.46
Medical ward		38	52574	0.072279	722.7914	4.69
Surgical ward		6	2631	0.228035	2280.351	4.34
Intensive Care Unit		236	68223	0.345922	3459.219	4.20
Head and neck (nerve) tumor center		94	57209	0.164308	1643.085	4.44
Department of eye, otolaryngology, head and neck surgery		26	20859	0.124647	1246.467	4.52
Department of traditional Chinese medicine		29	24370	0.119	1190	4.54
Emergency Department		384	339255	0.113189	1131.892	4.55
Outpatient Department		20	544980	0.00367	36.69863	5.47
Other departments^a^		13	6285	0.206842	2068.417	4.37
Total		2214	2068074	0.107056	1070.56	4.57

^a^The “Other” group includes four departments (Health Examination Center: 4 errors; Clinical Trial Institution: 3; Hyperbaric Oxygen Unit: 4; Gamma Knife Unit: 2)

**Table 3:** Hierarchical Clustering Characteristics.

Cluster	Error Rate	Sigma	Key Wards	n
1 (Excellence)	0.003-0.12%	4.52-5.47	Outpatient department Neurology W6/W8/W3/W5	13
2 (Standard)	0.13-0.17%	4.44-4.5	Neurology W7/W9, Neurosurgery W5/W6/W7 Head and neck (nerve) tumor center	10
3 (Watchlist)	0.21-0.27%	4.29 −4. 37	Neurosurgery W3/W11 Surgical ward	4
4 (Critical)	0.35-0.43%	4.13-4.20	Neurosurgery W1/W2 Intensive Care Unit	3

Notably, the Outpatient department, Neurology Wards 6, 8 and 3 demonstrated excellent performance with Sigma values exceeding 4.7. In contrast, Neurosurgery Wards 1 and 2, along with the ICU, showed suboptimal results (Sigma ≤ 4.2) and require immediate quality improvement measures. Chi-square tests confirmed that the Outpatient department’s performance was significantly superior to all other wards (*p* < 0.001).

### Quarterly trends of specimen rejection

**Fig 2:**
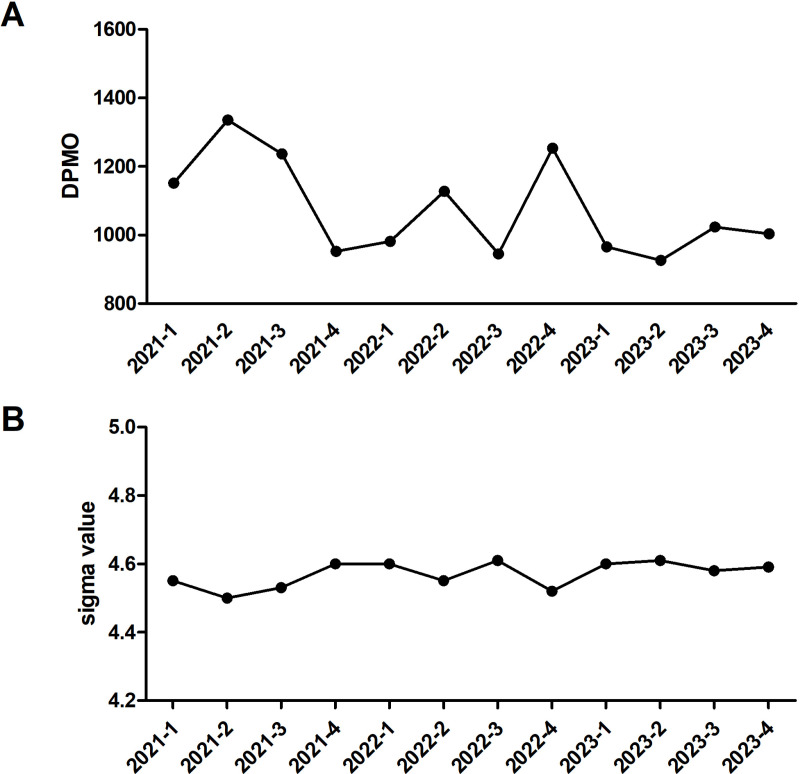
Quarterly trends of specimen rejection. Quarterly trends in specimen rejection rates from January 2021 to December 2023. (A) Specimen rejection rates expressed as defects per million opportunities (DPMO). (B) Corresponding Sigma values calculated based on DPMOs, illustrating changes in quality performance over time.

From 2021 to 2023, our analysis revealed a significant downward trend in specimen rejection rates (DPMO), despite periodic fluctuations (Fig 2A). The most substantial improvement occurred between 2021 and 2023, with ANOVA confirming significant quarterly variations (*p* = 0.017). Notably, Q4 2022 represented a marked exception to this trend, showing a 32.7% (1253.84 vs. 944.83 DPMO) increase in rejection rates compared to Q3 2022 (*p* = 0.038). This deviation was further validated by a parallel decline in Sigma values (4.61 to 4.52, Fig 2B), reinforcing the well-established inverse correlation between these quality indicators.

By 2023, rejection rates had stabilized within an improved range (926.66–1023.56 DPMO), suggesting successful implementation of sustained quality control measures. Our examination of seasonal patterns (excluding 2022 data) demonstrated that Q4 rates were consistently lower than Q3 by an average of 12.5% (2021: 1236.72 vs. 952.17; 2023: 1023.56 vs. 1003.99 DPMO). The observed seasonal variation did not reach statistical significance (*p* > 0.05), which may be attributed to the limited observational period. Nevertheless, the consistent directional trend merits consideration in future quality management planning.

### Proportional analysis of error types

**Fig 3:**
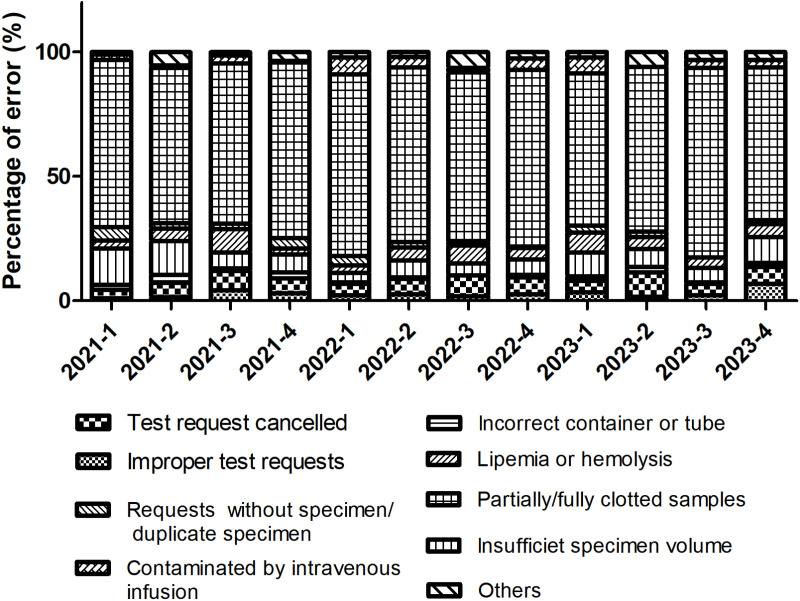
Proportional analysis of error types. Proportional distribution of specimen rejection types over the study period. Clotted blood samples were the most frequent source of pre-analytical errors throughout the three-year investigation.

Analysis of pre-analytical errors spanning 12 quarters from 2021 to 2023 revealed that “ clotted sample “ persistently dominated all error types, the quarterly proportion consistently remained within a high range above 60% (61.14%−76.32%), with a three-year average reaching 67.73% (Fig 3). Wilcoxon signed-rank test confirmed this was significantly higher than the combined proportion of all other error types (*p* < 0.001). Although the lowest value occurred in Q1 2023 (61.14%), it still exceeded sixty percent.

## Discussion

Effective pre-analytical phase management is essential for laboratory quality. Our laboratory rigorously monitors the four mandatory pre-analytical quality indicators (specimen type, container, volume, and clotting errors) established by China’s National Health Commission [7], supplemented by additional quality control indicators to ensure testing reliability. Our laboratory achieved a specimen rejection rate of 0.107% in this study, demonstrating superior pre-analytical quality compared to published studies. This performance is particularly noteworthy when compared with previously reported rejection rates: (1) the > 0.5% benchmark described by Simundic et al. as typical for many clinical laboratories [8], and (2) the 0.3% average observed across 78 facilities in Karcher and Lehman’s study [9]. Furthermore, broader analyses from large-scale investigations [10,11] collectively highlight the effectiveness of our quality control measures.

### Analysis of major error types

Clotted samples constituted the predominant pre-analytical error in our study, representing 67.34% of all rejections (Fig 3), consistent with neurological specialty hospital reports [10,12]. As highlighted by Lippi et al. [13], improper mixing of blood with anticoagulants is a major contributor to clotting errors, particularly in samples collected for routine blood tests. Similarly, Simundic et al. [8] emphasized that inadequate training of phlebotomy staff significantly increases the risk of clotting-related errors. Additionally, prolonged use of a pressure pulse belt during blood collection can accelerate coagulation. Clotting errors can be minimized through proper equipment, trained phlebotomists, and strict protocol adherence.

Insufficient specimen volume, including improper blood-to-anticoagulant ratios, accounted for 8.22% of rejection errors in our study (Table 1), representing the second most common cause of sample rejection. This rate compares favorably with the 22% reported by Jacobsz et al. [14]. Technical analysis revealed that volume errors primarily stemmed from vacuum system failures due to loose needle interfaces [15] or improper venipuncture angles creating false vacuum perception, premature needle withdrawal from inadequate endpoint training [16], syringe transfer inaccuracies, and anticoagulant ratio violations. As demonstrated by Bonini et al. [17], such volume errors significantly compromise test accuracy through either hemodilution (insufficient volume) or incomplete anticoagulation (excess volume). Hawkins’ work [18] further confirms these pre-analytical errors can increase laboratory turnaround times by 18–22%, underscoring the importance of our implemented solutions including mandatory pre-collection device checks, standardized withdrawal endpoint training, and automated volume verification systems.

Cancelled test requests accounted for 6.28% of specimen rejections in our study. Based on our institutional data, these cancellations predominantly involved three patient groups: discharged patients, deceased patients, and cases with difficult venous access. While such cancellations do not compromise test accuracy, they create unnecessary operational burdens. All healthcare providers (physicians and nursing staff) should make every effort to minimize such occurrences through careful patient assessment and order verification prior to specimen collection.

Additionally, the observed hemolysis/lipemia rate of 5.28% was lower than literature values [16], potentially reflecting detection limitations in our visual assessment protocol. This discrepancy may arise from: (1) undetected mild/moderate cases due to subjective visual evaluation, and (2) inability to differentiate in-vivo hemolysis from preanalytical causes [19]. Implementation of automated HIL indices would improve detection accuracy and enable proper classification of hemolytic causes, as demonstrated in recent studies [20].

### Quality assessment using sigma metrics

As Nevalainen [21] highlighted, laboratory quality indicators, often presented as percentages of variance, can be misleading and may appear deceptively low. In this study, the error rate for clotted samples was 0.173547%, indicating that 99.82645% of samples met the required standards. While this may seem like a satisfactory result at first glance, it is important to recognize that even a small percentage of a large sample size can represent a significant absolute number [21]. For instance, a Sigma value of 4.42 implies that out of one million samples received, approximately 1,735 may be clotted—a figure that is far from ideal. Therefore, expressing laboratory quality indicators on the Sigma scale provides a more rigorous and meaningful assessment of process performance. Low Sigma values underscore the need for targeted problem-solving strategies to refine processes and achieve sustained quality improvement. Our study achieved Sigma values >5.0 for all error types except clotted samples, this performance primarily reflects successful integration of two critical systems: (1) the barcode-enabled HIS that streamlined test ordering processes, and (2) the pneumatic transport network that reduced multiple specimen transport challenges, including delayed delivery, improper routing, and potential loss. Together, these technological solutions reduced human-dependent error sources that typically compromise pre-analytical quality, as documented in comparable laboratory automation studies [21].

### Departmental variations and temporal trends

The hierarchical clustering analysis effectively stratified clinical departments into distinct performance tiers based on specimen rejection patterns. Analysis revealed elevated specimen rejection rates in Neurosurgery Ward 1, 2 and ICU departments, primarily attributed to compromised collection standards during urgent procedures. To address this, we recommend implementing enhanced staff training on emergency specimen protocols and a continuous monitoring mechanism with monthly quality metrics tracking to identify and correct recurring issues. Notably, the superior performance of outpatient departments may reflect their standardized workflows and lower-acuity caseload, suggesting that process optimization strategies from these units could be adapted for inpatient settings.

The overall downward trend in specimen rejection rates from 2021 to 2023 demonstrates the effectiveness of our annual August training program in maintaining quality standards. This is particularly evidenced by the consistent seasonal pattern showing lower Q4 rejection rates compared to Q3 during typical operational years, suggesting the training yields measurable benefits in subsequent months.

The observed 32.7% increase in Q4 2022 rejection rates likely reflects the exceptional operational challenges during that period, including widespread staff infections and consequent workload intensification due to the COVID-19 outbreak. Nevertheless, the prompt return to improved performance levels in 2023 underscores both the resilience of our quality management system and the enduring value of consistent training protocols. These findings highlight the importance of maintaining robust training programs while developing contingency plans to address extraordinary circumstances.

### Limitations and improvement strategies

To build upon current quality achievements, further strategies are necessary to achieve sustained improvement. First, the development of rejection criteria with quantifiable thresholds (e.g., defined hemolysis indices, precise volume requirements) would reduce subjective decision-making while maintaining consistency. Second, establishing structured communication channels between laboratory and clinical teams enables collaborative resolution of recurrent preanalytical challenges and alignment on specimen collection requirements. Finally, periodic training programs, particularly for new staff, should be implemented to prevent avoidable errors and maintain high-quality standards.

While this study provides valuable insights into pre-analytical errors using Six Sigma metrics, several limitations should be acknowledged. First, the identification of hemolyzed and lipemic samples relied on visual assessment by technicians, as HIL indices were not measured. This subjective approach may have led to underestimation or overestimation of errors, particularly for mildly or moderately affected samples. Second, this study was conducted in a specialized brain hospital, and the sample types and handling protocols may differ significantly from those in general hospitals. This limits the broader applicability of our findings to other healthcare settings. Future studies should include diverse hospital settings to enhance the generalizability of the results. Finally, while we proposed potential strategies for quality improvement, such as staff training and the use of barcode systems, we did not systematically observe or quantify the effectiveness of these measures. Evaluating the impact of such interventions will be critical for guiding sustainable quality improvement efforts in the future.

## Supporting information

S1 Table Standard conversion table for calculating Sigma values. (XLS)

S2 Table Data of pre-analytical specimen rejection. Characteristics of pre-analytical specimen rejection, including occurrence time, rejection reasons, and error origins.
(XLSX)

S3 Table Summary of specimen numbers. Data including the total number of specimens, anticoagulated specimens, and ward-specific specimen counts during the same period of specimen rejection.
(XLS)

## References

[1] Ambachew S, Adane K, Worede A, Melak T, Asmelash D, Damtie S, et al. Errors in the total testing process in the clinical chemistry laboratory at the University of Gondar Hospital, Northwest Ethiopia. Ethiop J Health Sci. 2018;28(2):235–44. doi: 10.4314/ejhs.v28i2.15 29983521 PMC6016342

[2] Lippi G, Banfi G, Buttarello M, Ceriotti F, Daves M, Dolci A, et al. Recommendations for detection and management of unsuitable samples in clinical laboratories. Clin Chem Lab Med. 2007;45(6):728–36. doi: 10.1515/CCLM.2007.174 17579524

[3] Westgard JO, Westgard SA. The quality of laboratory testing today: an assessment of sigma metrics for analytic quality using performance data from proficiency testing surveys and the CLIA criteria for acceptable performance. Am J Clin Pathol. 2006;125(3):343–54. 16613337

[4] Teshome M, Worede A, Asmelash D. Total clinical chemistry laboratory errors and evaluation of the analytical quality control using sigma metric for routine clinical chemistry tests. J Multidiscip Healthc. 2021;14:125–36. doi: 10.2147/JMDH.S286679 33488088 PMC7815085

[5] Nevalainen D, Berte L, Kraft C, Leigh E, Picaso L, Morgan T. Evaluating laboratory performance on quality indicators with the six sigma scale. Arch Pathol Lab Med. 2000;124(4):516–9. doi: 10.5858/2000-124-0516-ELPOQI 10747306

[6] Harry M, Schroeder R. Six sigma: the breakthrough management strategy revolutionizing the world’s top corporations: Currency. 2000.

[7] Kang F, Li W, Xia X, Shan Z. Three years’ experience of quality monitoring program on pre-analytical errors in china. J Clin Lab Anal. 2021;35(3):e23699. doi: 10.1002/jcla.23699 33458892 PMC7958002

[8] Simundic A-M, Lippi G. Preanalytical phase--a continuous challenge for laboratory professionals. Biochem Med (Zagreb). 2012;22(2):145–9. doi: 10.11613/bm.2012.017 22838180 PMC4062337

[9] Karcher DS, Lehman CM. Clinical consequences of specimen rejection: a College of American Pathologists Q-Probes analysis of 78 clinical laboratories. Arch Pathol Lab Med. 2014;138(8):1003–8. doi: 10.5858/arpa.2013-0331-CP 25076290

[10] Bhat V, Tiwari M, Chavan P, Kelkar R. Analysis of laboratory sample rejections in the pre-analytical stage at an oncology center. Clin Chim Acta. 2012;413(15–16):1203–6. doi: 10.1016/j.cca.2012.03.024 22507083

[11] Deress T, Abebaw Y, Esayas Y, Nebertu S, Kinidie M, Abebe G, et al. Analyzing clinical laboratory specimen rejection rates at a specialized hospital in Ethiopia: a 2-year document review. Am J Clin Pathol. 2024;162(2):175–9. doi: 10.1093/ajcp/aqae019 38459898

[12] Guimarães AC, Wolfart M, Brisolara MLL, Dani C. Causes of rejection of blood samples handled in the clinical laboratory of a University Hospital in Porto Alegre. Clin Biochem. 2012;45(1–2):123–6. doi: 10.1016/j.clinbiochem.2011.10.009 22040813

[13] Lippi G, Salvagno GL, Montagnana M, Franchini M, Guidi GC. Phlebotomy issues and quality improvement in results of laboratory testing. Clin Lab. 2006;52(5–6):217–30. 16812947

[14] Jacobsz LA, Zemlin AE, Roos MJ, Erasmus RT. Chemistry and haematology sample rejection and clinical impact in a tertiary laboratory in Cape Town. Clin Chem Lab Med. 2011;49(12):2047–50. doi: 10.1515/CCLM.2011.743 21995606

[15] du Toit M, Chapanduka ZC, Zemlin AE. The impact of laboratory staff training workshops on coagulation specimen rejection rates. PLoS One. 2022;17(6):e0268764. doi: 10.1371/journal.pone.0268764 35657929 PMC9165799

[16] Lippi G, von Meyer A, Cadamuro J, Simundic A-M. Blood sample quality. Diagnosis (Berl). 2019;6(1):25–31. doi: 10.1515/dx-2018-0018 29794250

[17] Bonini P, Plebani M, Ceriotti F, Rubboli F. Errors in laboratory medicine. Clin Chem. 2002;48(5):691–8. 11978595

[18] Hawkins RC. Laboratory turnaround time. Clin Biochem Rev. 2007;28(4):179–94. 18392122 PMC2282400

[19] Lippi G, Plebani M, Di Somma S, Cervellin G. Hemolyzed specimens: a major challenge for emergency departments and clinical laboratories. Crit Rev Clin Lab Sci. 2011;48(3):143–53. doi: 10.3109/10408363.2011.600228 21875312

[20] Simundic A-M, Baird G, Cadamuro J, Costelloe SJ, Lippi G. Managing hemolyzed samples in clinical laboratories. Crit Rev Clin Lab Sci. 2020;57(1):1–21. doi: 10.1080/10408363.2019.1664391 31603708

[21] Hill PM, Mareiniss D, Murphy P, Gardner H, Hsieh Y-H, Levy F, et al. Significant reduction of laboratory specimen labeling errors by implementation of an electronic ordering system paired with a bar-code specimen labeling process. Ann Emerg Med. 2010;56(6):630–6. doi: 10.1016/j.annemergmed.2010.05.028 20822830

